# A general non-linear multilevel structural equation mixture model

**DOI:** 10.3389/fpsyg.2014.00748

**Published:** 2014-07-18

**Authors:** Augustin Kelava, Holger Brandt

**Affiliations:** Department of Education, Center for Educational Science and Psychology, Eberhard Karls Universität TübingenTübingen, Germany

**Keywords:** latent variables, semiparametric, non-linear, mixture distribution, structural equation modeling, multilevel

## Abstract

In the past 2 decades latent variable modeling has become a standard tool in the social sciences. In the same time period, traditional linear structural equation models have been extended to include non-linear interaction and quadratic effects (e.g., Klein and Moosbrugger, 2000), and multilevel modeling (Rabe-Hesketh et al., 2004). We present a general non-linear multilevel structural equation mixture model (GNM-SEMM) that combines recent semiparametric non-linear structural equation models (Kelava and Nagengast, 2012; Kelava et al., 2014) with multilevel structural equation mixture models (Muthén and Asparouhov, 2009) for clustered and non-normally distributed data. The proposed approach allows for semiparametric relationships at the within and at the between levels. We present examples from the educational science to illustrate different submodels from the general framework.

In the past 2 decades latent variable modeling has become a standard tool in the social sciences. Linear structural equation models have been extended to include non-linear interaction and quadratic effects (for a review see Schumacker and Marcoulides, 1998; Algina and Moulder, 2001; Marsh et al., 2004, 2006), and for the capability to model multilevel data structures (e.g., Rabe-Hesketh et al., 2004; Muthén and Asparouhov, 2009). However, a systematic combination of both non-linear structural equation modeling and multilevel modeling has not been implemented in a more general framework. In this article, we present a GNM-SEMM that combines recent semiparametric non-linear structural equation models (Kelava and Nagengast, 2012; Kelava et al., 2014) with multilevel structural equation mixture models (Muthén and Asparouhov, 2009) for clustered and non-Gaussian data. The proposed framework is capable of modeling non-linear parametric and semiparametric relationships at the within and at the between levels, and it allows non-normally distributed data to be considered. We will provide an empirical example from educational sciences to illustrate the applicability of the proposed framework. We will begin by providing an overview of current approaches for estimating non-linear structural equation models and current frameworks for multilevel structural equation (mixture) models.

## 1. Non-linear structural equation models

Numerous parametric approaches for the estimation of non-linear effects have been developed (for a review, see Schumacker and Marcoulides, 1998; Algina and Moulder, 2001; Marsh et al., 2004, 2006), including product indicator approaches (e.g., Kenny and Judd, 1984; Bollen, 1995; Jaccard and Wan, 1995; Ping, 1995; Jöreskog and Yang, 1996; Algina and Moulder, 2001; Marsh et al., 2004, 2006; Little et al., 2006; Kelava and Brandt, 2009), distribution analytic approaches (Klein and Moosbrugger, 2000; Klein and Muthén, 2007), Bayesian approaches (e.g., Arminger and Muthén, 1998; Lee et al., 2007), and method of moments based approaches (Wall and Amemiya, 2003; Mooijaart and Bentler, 2010). Whereas most product indicator approaches have been *ad-hoc* methods for the specification of non-linear interaction effects and have thus suffered from requiring complicated measurement models, recent distribution analytic and Bayesian approaches have tried to overcome the need for non-linear measurement models. Method-of-moments-based approaches (Wall and Amemiya, 2003; Mooijaart and Bentler, 2010) and some indicator approaches (Bollen, 1995; Jöreskog and Yang, 1996) have been proposed as methods that do not rely as heavily on the normality assumption of the latent variables as other approaches (e.g., the distribution analytic approaches). The relaxation of distributional assumptions may lead to a reduction in the threat of biased estimates for non-linear effects in situations in which data are non-normally distributed, but for most of these approaches, relaxing these assumptions is associated with a low power for detecting the effects (Schermelleh-Engel et al., 1998; Brandt et al., 2014).

A different approach for modeling non-linear relations between latent variables is the use of semiparametric structural equation mixture models (SEMM; Arminger and Stein, 1997; Jedidi et al., 1997a,b; Dolan and van der Maas, 1998; Arminger et al., 1999; Muthén, 2001; Bauer and Curran, 2004; Bauer, 2005; Pek et al., 2009, 2011). Finite mixtures of linear structural equation models are used to approximate the unknown functional form of the non-linear relationship of the latent variables^1^. Furthermore, by assuming mixtures, the SEMM approach relaxes the assumption of normally distributed latent variables and disturbances necessary in conventional structural equation models. Therefore, the SEMM approach is a flexible tool for predicting latent dependent variables when data are not normal, and when obtaining a strict parametric representation of the functional relation does not have the highest priority (for a discussion see Bauer, 2005). However, one drawback is that the linearity assumption of latent relationships and the normality assumption of the latent variables are relaxed simultaneously. This drawback can be manifested in the problem that observed non-normality in the data cannot be attributed to either non-normality of the latent variables or non-linearity between the latent variables. A way to overcome this problem is the specification of non-linear structural equation mixture models (NSEMM; Kelava et al., 2014) that allow distributional and linearity assumptions to be relaxed separately for the latent variables and their relationships.

Although, the use of mixtures for modeling non-linear latent variable relationships (e.g., Curran et al., 1996; Dolan and van der Maas, 1998; Bauer and Curran, 2004; Bauer, 2005) or the non-normality of latent variables in the context of non-linear structural equation models (Lubke and Muthén, 2005; Lee et al., 2008; Yang and Dunson, 2010; Kelava and Nagengast, 2012; Brandt et al., 2014; Kelava et al., 2014) have received increased attention in recent years, systematic evaluations have been rare. As an additional limitation, all approaches presented so far have been strictly limited to single-level models and have not accounted for nested data structures.

## 2. Multilevel structural equation modeling

Nested data structures have been addressed with multilevel models for relationships between manifest variables (for an introduction see Snijders and Bosker, 1999; Hox, 2010). In the past 2 decades, researchers have proposed frameworks that are capable of modeling nested data structures in latent variable models (e.g., Muthén, 1994; Rabe-Hesketh et al., 2004; Muthén and Asparouhov, 2009). For example, these frameworks have included models that account for random effects on the within-level, multilevel path analysis (Heck and Thomas, 2000), or multilevel confirmatory factor analysis (Muthén, 1994). Furthermore, mixtures of distributions have been applied in latent growth curve modeling (Muthén and Asparouhov, 2009).

So far, very limited psychometric developments have been proposed in the context of non-linear multilevel structural equation models that incorporate latent interaction effects. Leite and Zuo (2011) presented a product-indicator-based approach that allows for a specification of latent interactions on the between-level (e.g., at the school level). Their approach was a first attempt to extend the product-indicator approach for non-linear interaction effects in latent multilevel models. Products of between-level indicators are used for the specification of a measurement model of the between-level latent product variable.

Focusing more generally on within-person processes in psychology (Molenaar, 2004; Molenaar and Campbell, 2009), Nagengast et al. (2013) adapted the unconstrained product indicator approach to account for latent interactions on the within-level. In predicting homework motivation, they found support for the latent interaction between homework expectancy and homework value at the within-student level.

Despite these first successful adaptations, several problems that are associated with single-level non-linear structural equation modeling remain unsolved. First, the hitherto applied constrained and unconstrained product-indicator approaches for multilevel models are vulnerable to violations of distributional assumptions (normal distributions are typically assumed; for a discussion see Kelava et al., 2011). The specification of constrained and unconstrained product-indicator approaches strongly depends on the distributions involved (Kelava and Brandt, 2009), and biased estimates of the parameters and standard errors can be expected when specification errors occur (Kelava et al., 2008) or distributional assumptions are not met (Kelava and Nagengast, 2012). Hence, product-indicator approaches that are extended for multilevel data structures are even more vulnerable because more distributional assumptions on different levels have to be met.

Second, the proposed extensions of single-level non-linear structural equation models specify a parametric non-linearity (by involving products of latent variables). Recently, a strong emphasis has been placed on the relaxation of this simple functional relationship, including mixtures of latent variables that also allow for non-normally distributed variables (e.g., Bauer, 2005; Kelava et al., 2014). Therefore, on the one hand there is a need for an optional specification of a semiparametric relationship of the latent variables (at the within and between levels) to better approximate the non-linear reality. On the other hand, there is a need for an optional specification of mixtures that can account for non-normality or heterogeneity across subpopulations.

Third, the application of single-level non-linear structural equation modeling in substantive research has suffered from too many approaches that use the same distributional assumptions (see paragraphs above) and too few simulation studies that offer clear recommendations for the application of specific approaches (for an overview, see Kelava et al., 2011). Approaches that agree with regard to distributional assumptions may lead to contradictory results; that is, some approaches might suggest significant non-linear effects, whereas others might not. Substantive researchers cannot solve this kind of problem by referring to empirical data. Further information that is based on simulation studies (for single-level non-linear models see e.g., Brandt et al., 2014) is needed here.

In total, there is a need for a framework that incorporates several special cases of multilevel modeling and that offers general as well as specific solutions for both substantive and methodological research in non-linear latent variable modeling. From a substantive standpoint, non-linear hypotheses (e.g., interactions) can be examined in more detail. From a methodological standpoint, the framework will foster the comparison of different kinds of estimators (e.g., MCMC, ML, or moment methods) in the context of different distributions.

As a result of these considerations, in the next section, we will present a general non-linear multilevel structural equation mixture modeling (GNM-SEMM)framework that allows for the separate relaxation of distributional and linearity assumptions of the latent variables and their relationships on different levels of a nested data structure. We will provide several theoretical and practical examples to illustrate what is possible within the framework. In general, within this framework, it is possible to derive specific submodels that include crucial parts of the model as well as a combination of several aspects that have not been combined before.

## 3. A general non-linear multilevel structural equation mixture model

In this section, we will present a GNM-SEMM framework that allows for semiparametric latent non-linear effects on the within and the between levels. The framework presented here is similar to the general multilevel mixture model and notation presented by Muthén and Asparouhov (2009). Whereas Muthén and Asparouhov's (2009) model focuses only on linear relationships, the GNM-SEMM framework accounts for non-linear semiparametric relationships of the manifest and latent variables involved. This allows for a more precise modeling of latent variable relationships at different data levels while relaxing the linearity assumptions of standard latent multilevel frameworks (e.g., Rabe-Hesketh et al., 2004).

### 3.1. Observed and mixture variables

#### 3.1.1. Definition

Let *y*_*jik*_ be the score of the *j*-th (*j* = 1, …, *J*) observed (indicator) variable for individual *i* (*i* = 1, …, *N_k_*) in a cluster *k* (*k* = 1, …, *K*). Note that the individual index *i* is cluster-specific. Its range depends on the cluster size *N_k_* (e.g., the number of pupils in a given school *k* is denoted as *N_k_*). Let *z_lk_* be the score of the *l*-th (*l* = 1, …, *L*) observed (indicator) variable for cluster *k*. The observed scores *y*_*jik*_ and *z_lk_* could be realizations of dichotomous, ordered categorical, continuous normally distributed, or count variables.

Categorical (mixture) variables are used for the definition of mixtures on the individual (within) and cluster (between) levels. Let *C*_*ik*_ be an within-level latent categorical variable for individual *i* in cluster *k*, which takes values 1, …, C^*^_d_. Let *D_k_* be a between-level latent categorical variable for cluster *k*, which takes values 1, …, D^*^. Note that the number of latent classes on the within-level may be different across the latent classes on the between-level.

Analogous to Rabe-Hesketh et al. (2004), Muthén (1984), and Muthén and Asparouhov (2009), for observed dichotomous and ordered categorical variables, the underlying normally distributed latent variables y^*^_jik_ and z^*^_lk_ are defined such that for a set of threshold parameters τ_*jscd*_ and τ_*ls*′*d*_, and categories *s* and *s*′, respectively, the following equations hold for each subject *i* in cluster *k*:

(1) $$y_{jik}\;=s|{\;}_{C_{ik}=c,D_{k}\;=\;d}↔\tau_{jscd}<y_{jik}^{*}<\tau_{j,s\;+\;1,cd}$$

(2) $$z_{lk}\;=s^{\prime }{|}_{\;D_{k}\;=\;d}↔\tau_{ls^{\prime }d}<z_{lk}^{*}<\tau_{l,s^{\prime }\;+\;1,d,}$$

where the vertical bar ·|· indicates a “conditional on” statement, and ↔ indicates an equivalence. For continuous normally distributed variables, y^*^_jik_ = *y_jik_* and z^*^_lk_ = *z_lk_* are assumed, and for count variables, y^*^_jik_ = log(λ_*jik*_) and z^*^_lk_ = log(λ_*lk*_) hold, where λ_*jik*_ and λ_*lk*_ are the expectations of the Poisson distribution. Additional assumptions regarding the mean and covariance structure will be made in the following subsections, which will specify the measurement and structural models on the within and between levels.

#### 3.1.2. Example

**Figure 1:**
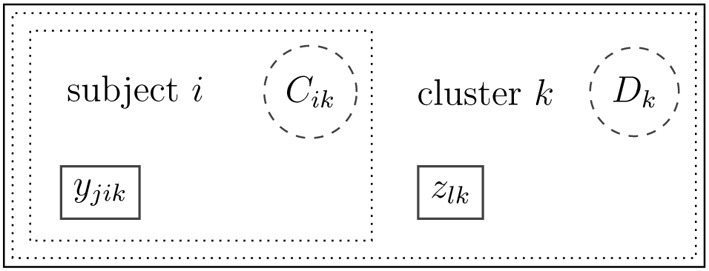
**Observed variable scores *y_jik_* (within-level) and *z_lk_* (between-level) as well as mixtures *C_ik_* (within-level) and *D_k_* (between-level)**.

Suppose that pupils from several schools take part in a math test. For a given pupil *i* from school *k* the score on a sub-task *j* from the math test is given by *y*_*jik*_. In addition, for school *k*, there is a score *z_lk_* that indicates the school's social problems (e.g., the degree of bullying reported by the principal). In Figure 1, two latent categorical variables *C*_*ik*_ and *D_k_* on the within-level (Level 1) and the between-level (Level 2), respectively, are introduced. These variables may account for heterogeneity that occurs in the scores on both levels. On Level 1, heterogeneity in the distribution of the math test may occur due to additional private lessons in math that some pupils received. On Level 2, heterogeneity may occur in the distribution of the school's social problems, for example, due to the general (unobserved) socioeconomic status of the neighborhood where the school is located. Furthermore, school *k* might belong to an unobserved group of schools *D_k_* = *d* that explicitly prepared for the math test. This may then influence the distribution of the math scores.

Figure 1 shows a diagram with the observed and mixture variables. At this stage, there is no model that can explain the relationship between the scores *y_jik_* and *z_lk_* and no measurement model that can describe the realizations of the scores. The mixtures are indicated by *C_ik_* and *D_k_*.

### 3.2. Level 1 – within level

#### 3.2.1. Measurement model

***3.2.1.1. Definition***. Let y^*^_ik_ be the *J*-dimensional vector for individual *i* in cluster *k* that includes scores for all dependent observed within variables. The measurement model is defined by a mixture distribution model

(3) $$y_{ik}^{*}{|}_{\;C_{ik}\;=\;c,D_{k}\;=\;d}\;=\nu_{1kcd}+\Lambda_{1kcd}f_{1}(\eta_{1ikcd})+K_{1kcd}g_{1}(x_{1ik})+\epsilon_{1ikcd}$$

where **ν**_1*kcd*_ is a *J*-dimensional vector of latent intercepts, **Λ**_1*kcd*_ is a *J* × *m*_(*f*_1_)_ loading matrix. **η**_1*ikcd*_ = (η_11*ikcd*_, …, η_1*ikmcd*_)′ is an *m*-dimensional vector of variables including all latent exogenous and endogenous variables. *f*_1_(·) is a smooth polynomial function mapping the *m*-dimensional variable vector **η**_1*ikcd*_ to an *m*_(*f*_1_)_-dimensional vector *f*_1_(**η**_1*ikcd*_). *f*_1_(**η**_1*ikcd*_) could be a vector that includes product variables [e.g., (η_11*ikcd*_, η_12*ikcd*_, η_11*ikcd*_ η_12*ikcd*_)′ or (η_11*ikcd*_, (η_11*ikcd*_)^2^, η_12*ikcd*_, (η_12*ikcd*_)^2^)′] (e.g., Schumacker and Marcoulides, 1998; Kelava et al., 2011) or splines (Freund and Hoppe, 2007). **K**_1*kcd*_ is a *J* × *Q*_(*g*_1_)_ matrix with regression coefficients. **x**_1*ik*_ is a *Q*-dimensional vector of all observed unexplained (within) covariates that may have an additional influence on the indicator variables y^*^_ik_. *g*_1_(·) is a smooth polynomial function mapping the *Q*-dimensional vector of covariates to a *Q*_(*g*_1_)_-dimensional vector *g*_1_(**x**_1*ik*_), and **ϵ**_1*ikcd*_ is a *J*-dimensional vector of residual variables with a zero mean vector and covariance matrix **Θ**_1*kcd*_.

For observed categorical variables **y**_*ik*_, a normality assumption for **ϵ**_1*ikcd*_ is equivalent to a probit regression for **y**_*ik*_ on **η**_1*ikcd*_ and **x**_1*ik*_. Alternatively, for dichotomous variables **y**_*ik*_, **ϵ**_1*ikcd*_ can have a logistic distribution, resulting in a logistic regression. For count variables **y**_*ik*_, the residual **ϵ**_1*ikcd*_ is assumed to be zero. For normally distributed continuous variables **y**_*ik*_, the residual variable **ϵ**_1*ikcd*_ is assumed to be normally distributed.

**Figure 2:**
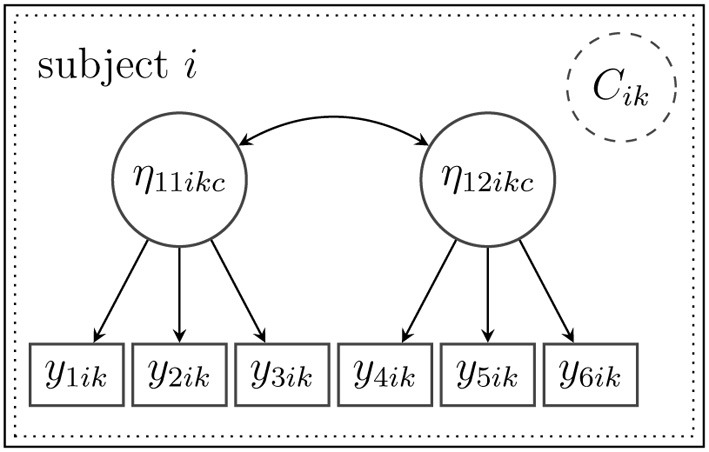
**A measurement model for subject *i* for two latent variables with a mixture distribution on the within-level (the between-level *i*th not included in this example)**. The mixture distribution is symbolized by the frame with dashed lines. It was assumed that all subjects belonged to one latent class *D* = 1 on the between-level so that the index *d* could be omitted.

***3.2.1.2. Example***. Suppose that in the above-mentioned math test example, data for two additional constructs (attitude toward reading and the teaching strategies experienced by the student) were collected with three items for each construct. The measurement model [cp. Equation (3)] is illustrated in Figure 2, and accordingly, it assumes two latent factors η_11*ikc*_ (attitude toward reading) and η_12*ikc*_ (experienced teaching strategies). For didactical purposes, all schools here belong to one class *D* = 1, so that the index *d* can be omitted, and there is no between-level model. Furthermore, heterogeneity is assumed on the within-level such that each pupil *i* belongs to an unobserved class (mixture) *C*_*ik* = *c*_. The example measurement model derived from the framework above is a confirmatory factor mixture model that is given by **y**_*ik*_|*_C_ik_ = c_* = **ν**_1*kc*_ + **Λ**_1*kc*_**η**_1*ikc*_ + **ϵ**_1*ikc*_. The heterogeneity, which is implied by the mixture *c*, can be accounted for differently by the (statistical) model depending on the hypothesized population model: First, a non-normal distribution of the latent variables can be modeled as a mixture distribution. For example, attitude toward reading might not be normally distributed. A mixture distribution of η_11*ikc*_ (with varying expectations and covariance structure for each mixture component *c*) could represent the non-normality (see Kelava et al., 2014). Second, the measurement model might be completely different for each unobserved subgroup (with varying factor loadings etc.). For example, some pupils might have poor reading skills, and hence, do not understand the items well enough. As a consequence, factor loadings in this subgroup may be lower (or residual variances may be larger) compared with other subgroups. and such differences may lead in turn to an observed heterogeneity.

#### 3.2.2. Structural model

The structural model for the latent variable vector **η**_1*ikcd*_ is given for each subject *i* in cluster *k* by

(4) $$\eta_{1ik}{|}_{\;C_{ik}\;=\;c,D_{k}\;=\;d}\;=\alpha_{kcd}+B_{1kcd}F_{1}(\eta_{1ikcd})+\Gamma_{1kcd}G_{1}(x_{1ik})+\zeta_{1ikcd}$$

where **α**_*kcd*_ is an *m*-dimensional vector of intercepts, **B**_1*kcd*_ is an *m* × *m*_(*F*_1_)_ loading matrix. *F*_1_(·) is a smooth polynomial function mapping the *m*-dimensional vector of latent variables **η**_1*ikcd*_ to an *m*_(*F*_1_)_-dimensional vector *F*_1_(**η**_1*ikcd*_). **Γ**_1*kcd*_ is an *m* × *Q*_(*G*_1_)_ matrix with regression coefficients. *G*_1_(·) is a smooth polynomial function mapping the *Q*-dimensional vector of covariates **x**_1*ik*_ to a *Q*_(*G*_1_)_-dimensional vector *G*_1_(**x**_1*ik*_). Note that for identification purposes, vector *G*_1_(**x**_1*ik*_) has to be completely different from vector *g*_1_(**x**_1*ik*_). **ζ**_1*ikcd*_ is an *m*-dimensional vector of residual variables with zero mean vector and covariance matrix **Ψ**_1*kcd*_.

#### 3.2.3. Mixture part

The model for the latent categorical variable *C_ik_* is a multinomial logit model

(5) $$Pr(C_{ik}=c|D_{k}=d,x_{1}=x_{1ik})\;=\frac{\exp\text{ ​}(a_{1kcd}+b_{1kcd}^{\prime }h_{1}(x_{1ik}))}{\sum_{t}\exp\;(a_{1ktd}+b_{1ktd}^{\prime }h_{1}(x_{1ik}))\text{​}}$$

where *a*_1*kcd*_ and **b**_1*kcd*_ are regression coefficients, and *h*_1_(·) is again a smooth (e.g., polynomial) function.

**Figure 3:**
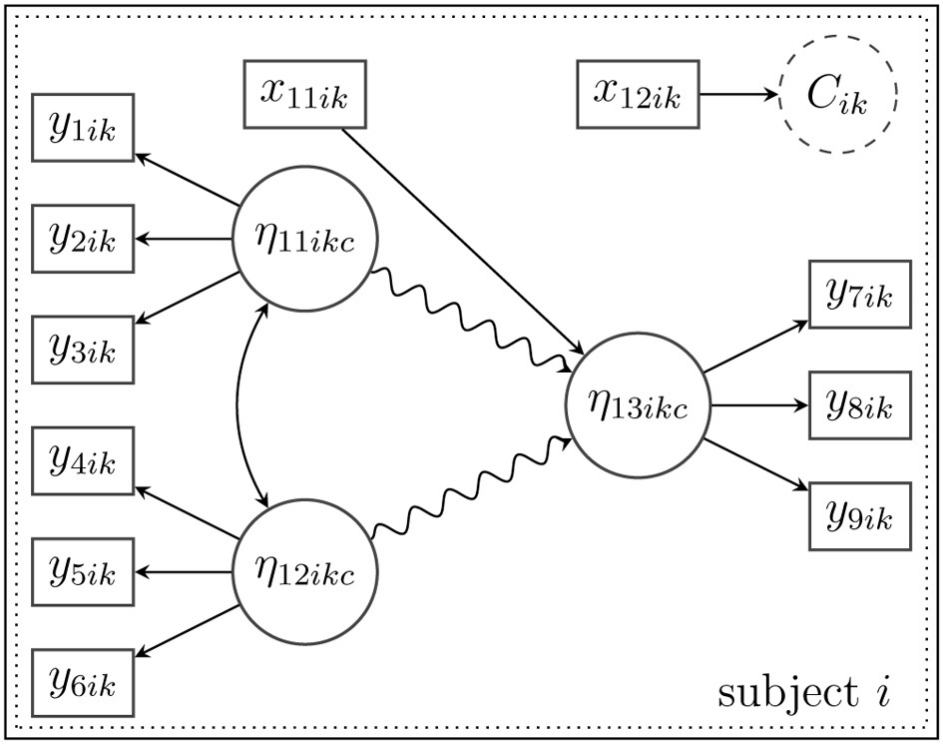
**Structural model for subject *i* in latent class *C_ik_* with a nonlinear spline relationship between the latent variables (indicated by the snake-type arrow)**. Note that this figure shows only a single-level model; the index *d* is therefore omitted.

***3.2.3.1. Example***. In the following illustrative example, the math skills of pupil *i* from school *k* (η_13*ikc*_) are predicted by the attitude toward reading (η_11*ikc*_) and by experienced teaching abilities (η_12*ikc*_; see also the example above). All three constructs are modeled as latent variables, which are measured with three indicator variables each. In addition, we assume that math skills can be predicted by gender, which is introduced into the model as an observed covariate (*x*_11*ik*_). For simplicity, the model is restricted to the within-level. Furthermore, it is assumed that there is unobserved heterogeneity due to a latent class *C_ik_*. Membership in one of the latent classes is predicted by a second observed covariate *x*_12*ik*_ (e.g., additional private math lessons). In contrast to an ordinary linear approximation of the relationship between the latent variables, the unknown and potentially curvilinear relationship is approximated by a latent spline model. Figure 3 illustrates the proposed model; the semiparametric spline model is indicated by the snake-type arrow.

### 3.3. Level 2 – between (cluster) level

The multilevel (between) part of the model is conceptualized as follows. Each of the intercepts (**ν**_1*kcd*_, **α**_*kcd*_, *a*_1*kcd*_) and slopes or loading parameters (**Λ**_1*kcd*_, **K**_1*kcd*_, **B**_1*kcd*_, **Γ**_1*kcd*_, **b**_1*kcd*_) in Equations (3), (4), and (5) can be either a fixed coefficient or a random effect that varies across the observed clusters *k*.

#### 3.3.1. Structural model

Let **η**_2*kd*_ be the *U*-dimensional vector of all such random effect variables and any additional between-level latent exogenous variables that explain these random effects and vary across the clusters. Note that **η**_2*kd*_ is different from **η**_1*ikcd*_ which is the individual-level latent variable vector. For a given cluster *k*, the between-level structural model for **η**_2*kd*_ is defined as

(6) $$\eta_{2k}{|}_{\;D_{k}\;=\;d}\;=\mu_{d}+B_{2d}F_{2}(\eta_{2kd})+\Gamma_{2d}G_{2}(x_{2k})+\zeta_{2kd}$$

where **μ**_*d*_ is a *U*-dimensional vector of intercepts, and **B**_2*d*_ is a *U* × *U*_(*F*_2_)_ loading matrix. *F*_2_(·) is a smooth polynomial function mapping the *U*-dimensional vector of variables **η**_2*kd*_ to a *U*_(*F*_2_)_-dimensional vector *F*_2_(**η**_2*kd*_). **Γ**_2*d*_ is a *U* × *V*_(*G*_2_)_ matrix with regression coefficients. **x**_2*k*_ is a *V*-dimensional vector of all observed unexplained between-level covariates that may have an additional influence on the variables in vector **η**_2*kd*_. Note that **x**_2*k*_ is different from **x**_1*ik*_. *G*_2_(·) is a smooth polynomial function mapping the *V*-dimensional vector of between-level covariates **x**_2*k*_ to a *V*_(*G*_2_)_-dimensional vector *G*_2_(**x**_2*k*_). **ζ**_2*kd*_ is a *U*-dimensional vector of residual variables with a zero mean vector and covariance matrix **Ψ**_2*d*_. **μ**_*d*_, **B**_2*d*_, and **Γ**_2*d*_ are fixed parameters.

**Figure 4:**
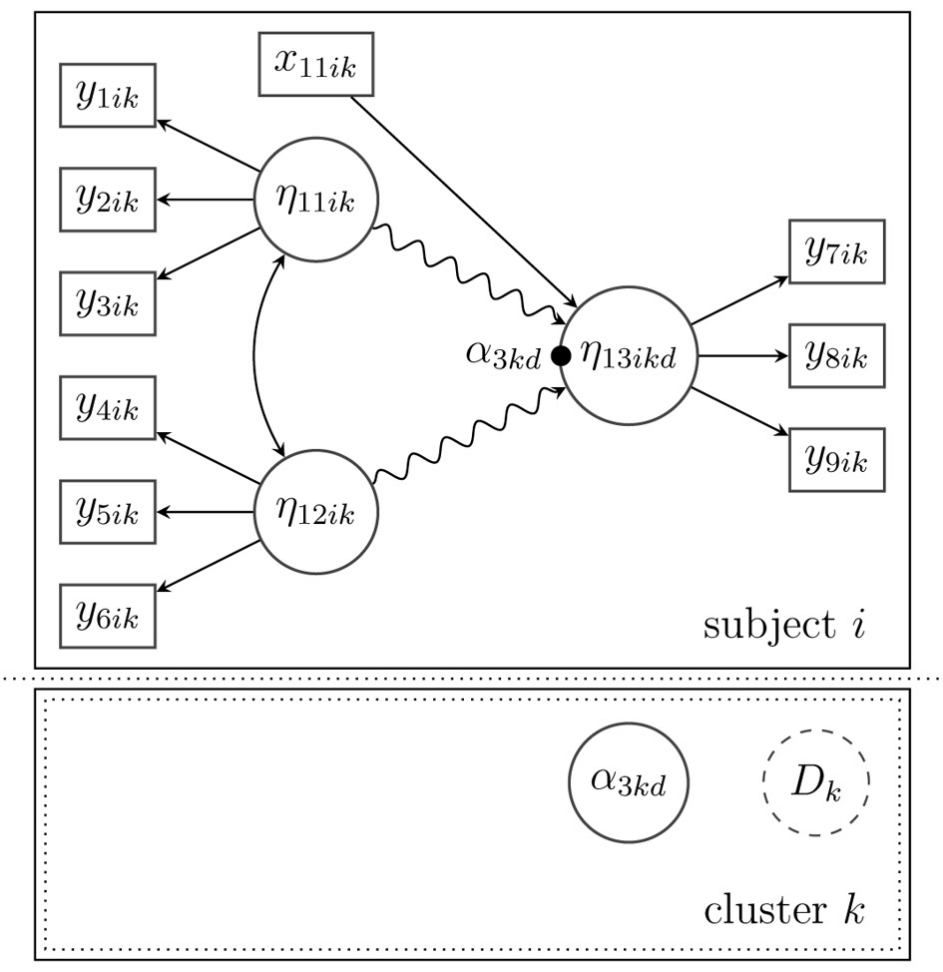
**Structural model for subject *i* in cluster *k* with a nonlinear spline relationship between the latent variables on the within-level (indicated by the snake-type arrow) and a random intercept (α_3*kd*_) that is modeled as a mixture of normal distributions on the between-level**.

***3.3.1.1. Example***. Suppose that the model in Figure 3 is extended to allow for multilevel effects on the between-level (Level 2). In Figure 4 depicts a latent random intercept model that implies a school-specific intercept (α_3*kd*_) for school *k* when the math skills (η_13*ikd*_) of a given pupil *i* are examined. In order to approximate a potentially non-normal distribution of the school-specific intercepts or to reveal a certain heterogeneity in the latent intercepts (i.e., average math skills), a latent mixture model with the latent categorical variable *D_k_* is applied. This mixture reflects Level-2 heterogeneity that may stem from (unobserved) sources, for example, certain school characteristics that influence the average math skills in school *k*.

#### 3.3.2. Measurement model

Let z^*^_k_ be the *L*-dimensional vector for cluster *k* that includes scores on all observed between-level variables that are indicators of the latent variables in vector **η**_2*kd*_. For a given cluster *k*, the measurement model is defined by

(7) $$z_{k}^{*}{|}_{\;D_{k}\;=\;d}\;=\nu_{2d}+\Lambda_{2d}f_{2}(\eta_{2kd})+K_{2d}g_{2}(x_{2k})+\epsilon_{2kd}$$

where **ν**_2*d*_ is an *L*-dimensional vector of intercepts, **Λ**_2*d*_ is an *L* × *U*_(*f*_2_)_ loading matrix. *f*_2_(·) is a smooth polynomial function mapping the *U*-dimensional vector of variables **η**_2*kd*_ to a *U*_(*f*_2_)_-dimensional vector *f*_2_(**η**_2*kd*_). **K**_2*d*_ is an *L* × *V*_(*g*_2_)_ matrix with regression coefficients. **x**_2*k*_ is the *V*-dimensional vector of all observed unexplained between-level covariates that may have an additional influence on the indicator variables z^*^_k_. *g*_2_(·) is a smooth polynomial function mapping the *V*-dimensional vector of between-level covariates **x**_2*k*_ to a *V*_(*g*_2_)_-dimensional vector *g*_2_(**x**_2*k*_). Note that for identification purposes *g*_2_(**x**_2*k*_) has to be completely different from *G*_2_(**x**_2*k*_). **ϵ**_2*kd*_ is a *L*-dimensional vector of residual (mixture) variables with a zero mean vector and covariance matrix **Θ**_2*d*_. **ν**_2*d*_, **Λ**_2*d*_, and **K**_2*d*_ are fixed parameters.

#### 3.3.3. Mixture part

The model for the between-level categorical variable *D_k_* is also a multinomial logit regression

(8) $$Pr(D_{k}=d|x_{2}=x_{2k})\;=\frac{\exp\text{ ​}(a_{2d}+b_{2d}^{\prime }h_{2}(x_{2k}))}{\sum_{t}\exp\;(a_{2t}+b_{2t}^{\prime }h_{2}(x_{2k}))\text{​}}$$

where *a*_2*d*_ and **b**_2*d*_ are regression coefficients, and *h*_2_(·) is again a smooth (e.g., polynomial) function.

**Figure 5:**
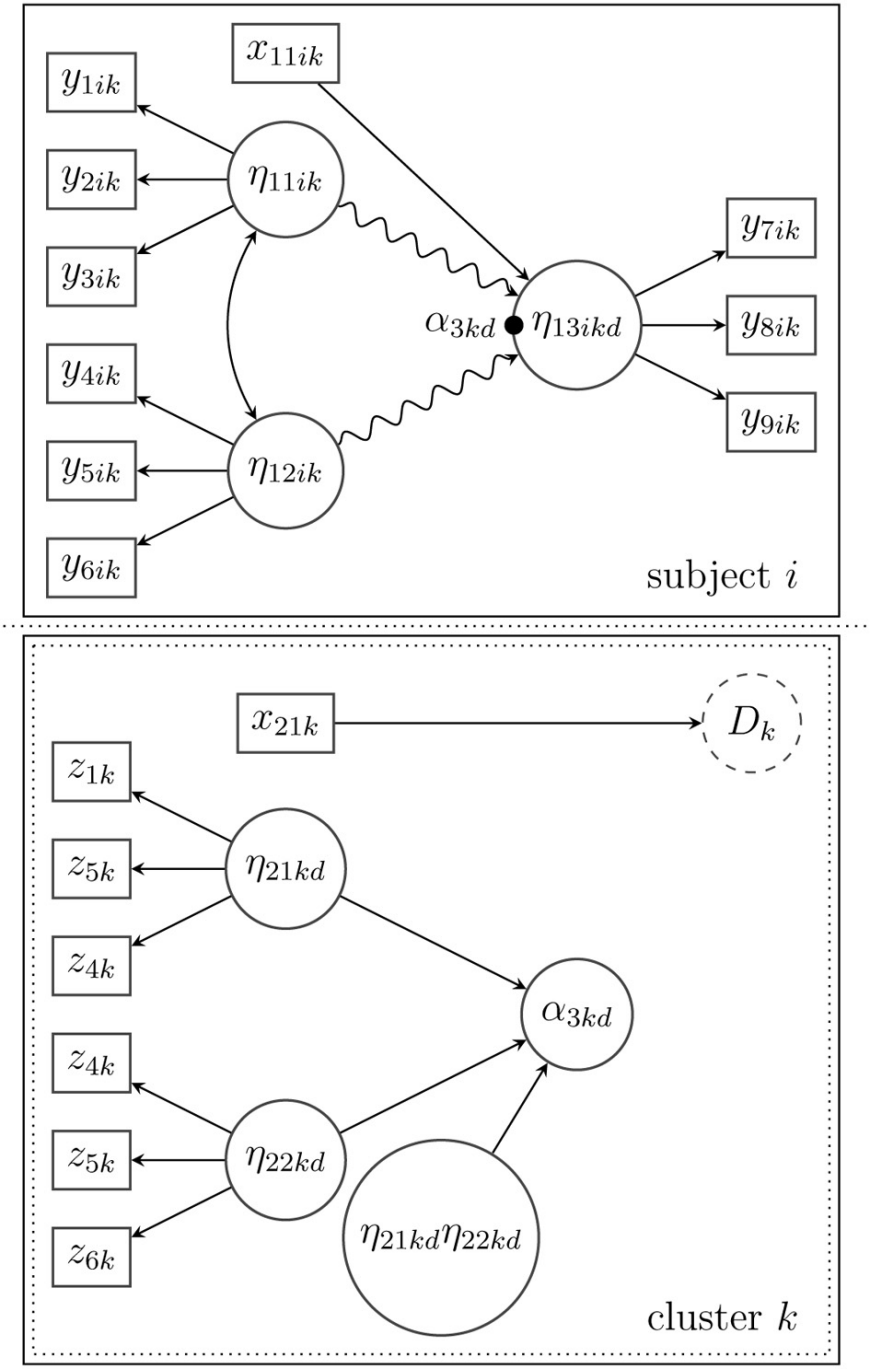
**Structural model for subject *i* in cluster *k* with a spline relationship between the latent variables on the within-level (indicated by the snake-type arrow), and a random intercept (α_3*kd*_) that is predicted by an interaction model on the between-level**. The distribution of the between-level's predictors is approximated by a mixture of normal distributions. The latent categorical variable *D_k_* is predicted by a between-level covariate *x*_21*k*_.

***3.3.3.1. Example***. In this last example (see Figure 5, the random intercept model in Figure 4 has been expanded by adding two latent Level-2 predictor variables (η_21*kd*_ and η_22*kd*_) that may influence the average math-skill level, for example, structural problems and social problems in school. Besides the linear effects of the latent predictors, there is an interaction effect that models the hypothesis that high scores on both between-level predictors may lead to a particularly low (or high) average math-skill level. A potential heterogeneity of the latent predictors (e.g., a non-normal distribution) is taken into account by introducing a latent categorical variable *D_k_*. In addition, a manifest predictor variable *x*_21*k*_, for example, school size or school type (private or public), is included in the model to predict the latent class probability of *D_k_* as described more generally in Equation (8).

### 3.4. Summary

In the model described by Equations (3) to (8), the latent variables on Level 1 (**η**_1*ikcd*_, **ϵ**_1*ikcd*_, and **ζ**_1*ikcd*_) and on Level 2 (**η**_2*kd*_, **ϵ**_2*kd*_, and **ζ**_2*kd*_) are conceptualized as variables stemming from mixtures on level 1 and level 2, respectively. The possibility of specifying within- and between-level mixture components is a result of introducing the latent categorical variables *C_ik_* and *D_k_* on the individual and cluster levels, respectively. On the within-level, unobserved latent classes may refer to different subpopulations (within each cluster), for example, pupils with different socioeconomic backgrounds in a given school. On the between-level, latent mixtures additionally allow for a specification of heterogeneity across/between observed clusters, for example, heterogeneity that is caused by certain characteristics of the schools. Furthermore, due to the conceptualization of mixture variables, a semiparametric modeling of non-normally distributed latent variables is available (e.g., Yang and Dunson, 2010; Kelava and Nagengast, 2012; Kelava et al., 2014), or a simple semiparametric formulation of the latent relationships (e.g., Bauer, 2005) is possible. Finally, the implementation of general polynomial functions *F*_1_(·), *f*_1_(·), *G*_1_(·), and *g*_1_(·) allows for a flexible inclusion of different parametric or semiparametric relationships (e.g., interaction effects or splines; Hastie et al., 2009), which extends the opportunities to model non-linear effects (e.g., Guo et al., 2012; Song et al., 2013).

## 4. Model identification

As in any other latent variable framework, within the GNM-SEMM framework, the user must ensure that the specified model is identified. In this section, we will summarize important aspects that need to be considered even though model identification is not straightforward (cf. San Martín et al., 2011; Song et al., 2013). For the identification of the proposed model, four separate aspects need to be taken into account. However, the actual identification of a specific model needs to be examined individually.

First, within each mixture component standard assumptions for non-linear structural equation models need to be met. This mainly implies that restrictions be placed on manifest scaling variables or latent exogenous variables (e.g., a necessary condition for the identification is to set one factor loading for each latent predictor variable or the latent predictors' variance to one). In addition, either the latent intercepts of the indicator variables or the latent intercepts of the latent variables may be estimated in a model. Note that when latent exogenous variables (e.g., η_11*ikcd*_, η_12*ikcd*_) are identified, their latent product terms (e.g., η_11*ikcd*_ η_12*ikcd*_) do not need product indicators for identification (cf. Klein and Moosbrugger, 2000).

Second, regarding the inclusion of polynomial functions for the observed covariates, it is necessary that the vectors *g*_1_(**x**_1*ik*_) and *G*_1_(**x**_1*ik*_) on Level 1 and, respectively, the vectors *g*_2_(**x**_2*k*_) and *G*_2_(**x**_2*k*_) on Level 2 are completely different from each other. For example, a model including *g*_1_(**x**_1*ik*_) = *G*_1_(**x**_1*ik*_) = (*x*_11*ik*_, *x*^2^_11*ik*_)' would not be identified because *x*_11*ik*_ would be a predictor in the measurement and structural models [see Equations (3) and (4)]. In this case, two effects of *x*_11*ik*_ would be estimated simultaneously on the right side of one regression equation, which would not be identified. The same holds for the polynomial functions of the latent variables. Again, *f*_1_(**η**_1*ikcd*_) and *F*_1_(**η**_1*ikcd*_) on Level 1 as well as *f*_2_(**η**_2*kd*_) and *F*_2_(**η**_2*kd*_) on Level 2 have to be unequal [see Equations (7) and (6)]^2^. Otherwise, perfect collinearity would be the result, meaning that the covariates and latent variables, respectively, would have the same influence on the measurement and the structural models. Their impacts would not be separable. Furthermore, polynomial (semiparametric) functions should not include constants. Otherwise, latent intercepts in the measurement and structural models would not be identified.

Third, on the between (cluster) level the inclusion of latent exogenous variables, which explain the variability in the random coefficients, requires measurement models (see Figure 5). The exogenous latent variables at Level 2 need to be identified as well according to identification rules, which are the same as in single-level structural equation models.

Fourth, additional assumptions concerning the latent classes of the mixture components are required. For the identification of the discrete latent variables, (a) the unconditional probabilities in Equations (5) and (8) need to sum up to one. and (b), the ambiguity of mixture components due to the so-called label switching problem makes it necessary to impose additional (artificial) constraints or relabeling strategies e.g., restrictions on the mean structure or ordinality of mixture proportions (see Equations 15–19; Redner and Walker, 1984; Stephens, 2000; Kelava and Nagengast, 2012).

Note that the identification of separate parts of a model (e.g., the measurement model and the structural model) does not automatically imply that the whole model is identified. General necessary and sufficient conditions to guarantee the identifiability of a latent variable model are difficult to establish. Hence, we focus primarily on the necessary identification conditions in this article.

## 5. Model estimation

Generally speaking, latent variable modeling offers a large variety of methods for the estimation of specified models. The choice of the best estimation method strongly depends on the distributional assumptions of the observed and latent variables, the given sample size, the type of specified model, potential confounders, and many more aspects. Just to mention a few large classes, these methods comprise maximum likelihood estimators (e.g., Jöreskog, 1973; Rabe-Hesketh et al., 2005; Muthén and Asparouhov, 2009), least squares methods (e.g., Joreskog and Goldberger, 1972; Browne, 1974, 1984), and methods of moments (e.g., Bentler, 1983), among others. For example, when applying a maximum likelihood estimator, in the well-known EM algorithm (Dempster et al., 1977), which treats latent variables as missing data, the likelihood **L** of the observed indicator vector **y** is given as:

(9) $$\begin{array}{l} L=\prod_{k}\sum_{d}Pr(D_{k}=d)\int \psi_{2kd}(\eta_{2kd})\prod_{i}\\ \;\left(\sum_{c}Pr(C_{ik}=c)\int f_{1ikcd}(y_{ik})\psi_{1ikcd}(\eta_{1ikcd})d\eta_{1ikcd}\right)\;d\eta_{2kd} \end{array}$$

where *f*_1*ikcd*_(·), ψ_1*ikcd*_(·), and ψ_2*kd*_(·) are probability density functions for the observed variables **y**, and the latent variables **η**_1*ikcd*_ and **η**_2*kd*_, respectively (cf. Muthén and Asparouhov, 2009). Because the likelihood function **L** of the observed indicator vector **y**_*ik*_ is not given in closed form in general, numerical integration can be utilized in the evaluation of the likelihood using both adaptive and non-adaptive quadrature. As an alternative, the likelihood could be directly optimized by applying a quasi-Newton algorithm. Both approaches of estimating parameters are very complex due to the non-linearity (for a discussion of latent interaction effects, see Klein and Moosbrugger, 2000).

However, in recent years, the Bayesian framework has become very popular in latent variable modeling (e.g., Lee et al., 2004; Lee, 2007; Lee et al., 2007; Song et al., 2009). The main reason is that it provides flexible options for specifying and estimating models. Bayesian estimation methods and algorithms (e.g., Markov Chain Monte Carlo: MCMC) can handle numerous complex parametric, semiparametric, and non-parametric relationships and distributions, for example, latent mixture distributions (e.g., Yang and Dunson, 2010; Kelava and Nagengast, 2012), non-linear models (e.g., Lee et al., 2007; Guo et al., 2012; Song et al., 2013), and multilevel structures (e.g., Fox and Glas, 2001; Song and Lee, 2004). Referring to the proposed GNM-SEMM framework with its semiparametric functional forms and its capability of considering non-normally distributed variables, a Bayesian approach seems to be a viable way to estimate models. In this sense, we will provide general descriptions of the specifications of the variables' distributions and the selection of prior distributions.

Parameter vectors are defined as follows: For the Level-1 parameters, let θ_*M*1*kcd*_ = (**ν**′_1*kcd*_, *vec*(**Λ**_1*kcd*_)′, *vec*(**K**_1*kcd*_)′, *vec*(**Θ**_1*kcd*_)′)′ for the measurement model, where *vec*(·) vectorizes all elements of a given matrix. For the structural model, let θ_*S*1*kcd*_ = (**α**′_*kcd*_, *vec*(**B**_1*kcd*_)′, *vec*(**Γ**_1*kcd*_)′, *vec*(**Ψ**_1*kcd*_)′)′, and for the mixture model part let θ_*m*1*kcd*_ = (*a*_1*kcd*_, **b**′_1*kcd*_)′. Analogously, for the Level-2 parameters, let θ_*M*2*d*_ = (**ν**′_2*d*_, *vec*(**Λ**_2*d*_)′, *vec*(**K**_2*d*_)′, *vec*(**Θ**_2*d*_)′)′ for the measurement model. For the structural model, let θ_*S*2*d*_ = (**μ**′_*d*_, *vec*(**B**_2*d*_)′, *vec*(**Γ**_2*d*_)′, *vec*(**Ψ**_2*d*_)′)′, and for the mixture model part let θ_*m*2*d*_ = (*a*_2*d*_, **b**′_2*d*_)′. Finally, let θ_*M*1_, θ_*S*1_, θ_*m*1_, θ_*M*2_, θ_*S*2_, and θ_*m*2_ be the vectors that include all parameters from the defined model parts across all latent classes *c* = 1, …, C^*^_d_, *d* = 1, …, D^*^, and observed clusters *k* = 1, …, *K*.

### 5.1. Specification of the variables' distribution

#### 5.1.1. Level 1

For the Bayesian analysis, the *j* = 1, …, *J* indicator variables on Level 1 are specified as a cluster-specific mixture distribution. The single mixture is given as

(10) $$y_{ik}^{*}|{\;}_{\theta_{M1},\theta_{S1},x_{1ik},C_{ik}\;=\;c,D_{k}\;=\;d}\;\sim N(\mu^{y^{*}}(\theta_{M1kcd},\theta_{S1kcd},x_{1ik}),\;\Theta_{1kcd}^{-1})$$

where **μ**^y^*^^(θ_*M*1*kcd*_, θ_*S*1*kcd*_, **x**_1*ik*_) is the vector of conditional expectations of y^*^_ik_, which are specified in Equation (3) and depend on the parameter vectors θ_*M*1*kcd*_ and θ_*S*1*kcd*_, and on the covariate vector **x**_1*ik*_. **Θ**^−1^_1*kcd*_ is the precision matrix of the multivariate normal distribution of the measurement error variables (i.e., the inverse of the covariance matrix). The model implies a specific mean vector and covariance matrix for subjects stemming from a certain latent class *c* on Level 1 that is clustered in a latent class *d* on Level 2, which in turn is given for an observed cluster *k*. Within each cluster *k*, y^*^_ik_ is a mixture of D^*^ components, which model heterogenity in the observed clusters. Further, within in each mixture component *d*, y^*^_ik_ is a mixture of C^*^_d_ components, which induce heterogenity on the individual level (C^*^_d_ may change across different latent classes on Level 2).

The latent variables **η**_1*ikcd*_ on Level 1 are specified as

(11) $$\eta_{1ik}{|}_{\;\theta_{S1},x_{1ik},C_{ik}\;=\;c,D_{k}\;=\;d}\;\sim N(\mu^{\eta_{1}}(\theta_{S1kcd},x_{1ik}),\Psi_{1kcd}^{-1})$$

with the vector **μ**^η_1_^(θ_*S*1*kcd*_, **x**_1*ik*_) of conditional expectations for **η**_1*ikcd*_ that depend on the parameter vector θ_*S*1*kcd*_ and covariate vector **x**_1*ik*_ as specified in Equation (4) as well as in the precision matrix **Ψ**^−1^_1*kcd*_.

#### 5.1.2. Level 2

Analogous to the specification of the variables' distributions on Level 1, the indicator vector z^*^_k_ is modeled as

(12) $$z_{k}^{*}{|}_{\;\theta_{M2},\theta_{S2},x_{2k},D_{k}\;=\;d}\;\sim N(\mu^{z^{*}}(\theta_{M2d},\theta_{S2d},x_{2k}),\Theta_{2d}^{-1})$$

with the vector **μ**^z^*^^(θ_*M*2*d*_, θ_*S*2*d*_, **x**_2*d*_) of conditional expectations for z^*^_k_ as specified in Equation (7) and precision matrix **Θ**^−1^_2*d*_. The unconditional indicator vector z^*^_k_ is composed of D^*^ mixture components. Finally, the distribution of the latent variable vector **η**_2*kd*_, is given as

(13) $$\eta_{2k}{|}_{\;\theta_{S2},x_{2k},D_{k}\;=\;d}\;\sim N(\mu^{\eta_{2}}(\theta_{S2d},x_{2k}),\Psi_{2d}^{-1})$$

with the vector of conditional expectations **μ**^η_2_^(θ_*S*2*d*_, **x**_2*k*_) specified in Equation (6) and precision matrix **Ψ**^−1^_2*d*_.

### 5.2. Specification of prior distributions

For the prior specification, informative or non-informative priors can be selected (Gelman et al., 2004). This selection is primarily based on the availability of prior knowledge. Because the application of non-informative priors may lead to suboptimal solutions (e.g., Lee et al., 2007), it may be necessary to analyze parts of the model (e.g., a confirmatory factor analysis for the Level-2 predictors) to obtain information about the parameters. Here, a very general description of the proposed model is provided. For a detailed description of priors see Gelman et al. (2004).

The class probabilities Pr(*C_ik_* = *c*|*D_k_* = *d*, **x**_1*ik*_) and *Pr*(*D_k_* = *d*|**x**_2*k*_) depend on the multinomial logit models given in Equations (5) and (8) and thus depend on the parameters in θ_*m*1_ and θ_*m*2_. For these parameters, uninformative priors are suggested unless information about heterogeneity is available (see also Kelava and Nagengast, 2012).

For each precision matrix of the mixture distributions defined above, that is for **Θ**^−1^_1*kcd*_, **Θ**^−1^_2*d*_ for the indicator variables, and for **Ψ**^−1^_1*kcd*_, **Ψ**^−1^_2*d*_ for the latent variables, a multivariate normal distribution is assumed within each component. Conjugate priors are then given for *c* = 1, …, C^*^_d_, *d* = 1, …, D^*^ as

(14) $$\begin{array}{l} \Theta_{1kcd}^{-1}\sim W(\Theta_{01kcd}^{-1},\rho^{\Theta_{1kcd}})\\ \Theta_{2d}^{-1}\sim W(\Theta_{02d}^{-1},\rho^{\Theta_{2d}})\\ \Psi_{1kcd}^{-1}\sim W(\Psi_{01kcd}^{-1},\rho^{\Psi_{1kcd}})\\ \Psi_{2d}^{-1}\;\sim W(\Psi_{02d}^{-1},\rho^{\Psi_{2d}}). \end{array}$$

The hyperparameters ρ and the (positive definite) matrices **Θ**_**0**1*kcd*_, **Θ**_**0**2*d*_, **Ψ**_**0**1*kcd*_, and **Ψ**_**0**2*d*_ of the Wishart distribution include parameter information that may stem from previous studies or knowledge about the parameters. For example, **Ψ**_**0**_2*d* includes information about the variances and covariances of the random coefficients, and about the latent endogenous and exogenous variables on Level 2. This information may refer to estimates of the (co)variances for the latent exogenous variables retrieved from a separately estimated confirmatory factor analysis.

The conjugate priors can be modified, for example, if the residual covariance matrix **Θ**_2*d*_ on Level 2 is assumed to be diagonal, then each diagonal element Θ^*j*^_2*d*_ (*j* = 1, …, *J*) can be assumed to be inverse Gamma distributed, that is (Θ^*j*^_2*d*_)^−1^ ~ *Gamma*(α_Θ^*j*^_2*d*__, β_Θ^*j*^_2*d*__) (with hyperparameters α, β) (Kelava and Nagengast, 2012). Further information about the selection of priors for count or ordinal data can be found in Song et al. (2013).

For the other parameters in θ_*M*1_, θ_*S*1_, θ_*M*2_, and θ_*S*2_, normally distributed priors are used within each mixture component. Though, the definition of some priors needs to be formulated recursively (cf. Kelava and Nagengast, 2012). For example, let ν^*j*^_1*kcd*_ be the *j*-th element of the vector **ν**_1*kcd*_ (which specifies the intercept of the *j*-th variable in y^*^_ik_|_*C*_*ik*_ = *c*, *D_k_* = *d*_), and let Θ^*j*^_1*kcd*_ be the *j*-th diagonal element in the matrix **Θ**_1*kcd*_. Then for the latent classes *c* = 1, *d* = 1, the conjugate (normal) prior for ν^*j*^_1*k*11_ is specified as

(15) $$\nu_{1k11}^{j}|\Theta_{1k11}^{j}\sim N(\nu_{01k11}^{j},\Theta_{1k11}^{j}H_{0})$$

with hyperparameters **H**_0_ and ν^*j*^_**0**_1*k*11 that include information about ν^*j*^_1*k*11_. For all other latent classes, that is *c* > 1 or *d* > 1, the following prior is selected:

(16) $$\begin{array}{l} \nu_{1k1d}^{j}|\Theta_{1k1d}^{j}=\;\nu_{1k1(d-1)}^{j}|\Theta_{1k1(d-1)}^{j}+\;\Delta_{1k1(d-1)}^{\nu j}|\Theta_{1k1d}^{j}\\ \text{ if }c=1,d>1 \end{array}$$

(17) $$\begin{array}{l} \nu_{1kc1}^{j}|\Theta_{1kc1}^{j}=\nu_{1k(c-1)1}^{j}|\Theta_{1k(c-1)1}^{j}+\Delta_{1k(c-1)1}^{\nu j}|\Theta_{1kc1}^{j}\\ \text{ if }c>1,d=1 \end{array}$$

(18) $$\begin{array}{l} \nu_{1kcd}^{j}|\Theta_{1kcd}^{j}=\nu_{1k(c-1)(d-1)}^{j}|\Theta_{1k(c-1)(d-1)}^{j}+\Delta_{1k(c-1)(d-1)}^{\nu j}|\Theta_{1kcd}^{j}\\ \text{ else}, \end{array}$$

with

(19) $$\Delta_{1kcd}^{\nu j}|\Theta_{1kcd}^{j}\sim N(0,\Theta_{1kcd}^{j}H_{0})\text{, and }\Delta_{1kcd}^{\nu j}|\Theta_{1kcd}^{j}\in (0,\infty ).$$

If parameters are constrained to be the same across mixture components (e.g., **ν**_1*kcd*_ = **ν**_1*k*_ and **Θ**_1*kcd*_ = **Θ**_1*k*_), Equations (15) to (19) simplify to

(20) $$\nu_{1k}^{j}|\Theta_{1k}^{j}\sim N(\nu_{01k}^{j},\Theta_{1k}^{j}H_{0}).$$

For the other parameter matrices, that is for **Λ**_1*kcd*_, **K**_1*kcd*_, **α**_*kcd*_, **B**_1*kcd*_, **Γ**_1*kcd*_ and so forth on Level 1 and **ν**_2*d*_, **Λ**_2*d*_, **K**_2*d*_, **μ**_*d*_, **B**_2*d*_, **Γ**_2*d*_ and so forth on Level 2, a specification corresponding to the formulation above given is straightforward when the appropriate precision matrices are used. In order to avoid the label-switching problem in a mixture distribution, only one of the parameter matrices needs to be formulated recursively.

## 6. Empirical example

In this section, we will provide an extensive illustration of the GNM-SEMM with an example that is based on data from the Program for International Student Assessment 2009 (PISA; Organisation for Economic Co-Operation and Development, 2010), which is publicly available under http://pisa2009.acer.edu.au/downloads.php. The sample was a German subsample of *N* = 1, 474 pupils from 226 schools who took a math test. Additional covariate information were available on the individual level as well as on the school level.

As before, we predicted *pupil's math skills* (Math) with their *general attitude toward reading* (Att) and the *teaching strategies they experienced* (Strat). We further expected that pupil's average math skills (latent intercept of Math) would vary systematically across schools^3^, and that this variation could be (partly) accounted for by Level-2 predictors with measurement errors, here, *structural problems in school* (Prob) and the *schools's social environment* (Soc).

We will report the results for a model that accounted for different aspects of the general model. The example is not exhaustive with regard to all potential parameters within the GNM-SEMM framework, but it provides an indication of the flexibility of the proposed framework in accommodating different aspects of the data: A spline model on Level 1 described a semiparametric flexible relationship between Att, Strat, and Math. A random intercept for Math was explained by the Level-2 predictors Prob and Soc, and the interaction effect between them. Furthermore, a mixture model accounted for the non-normality of the latent predictors on Level 2 (heterogeneity).

### 6.1. Model formulation

**Figure 6:**
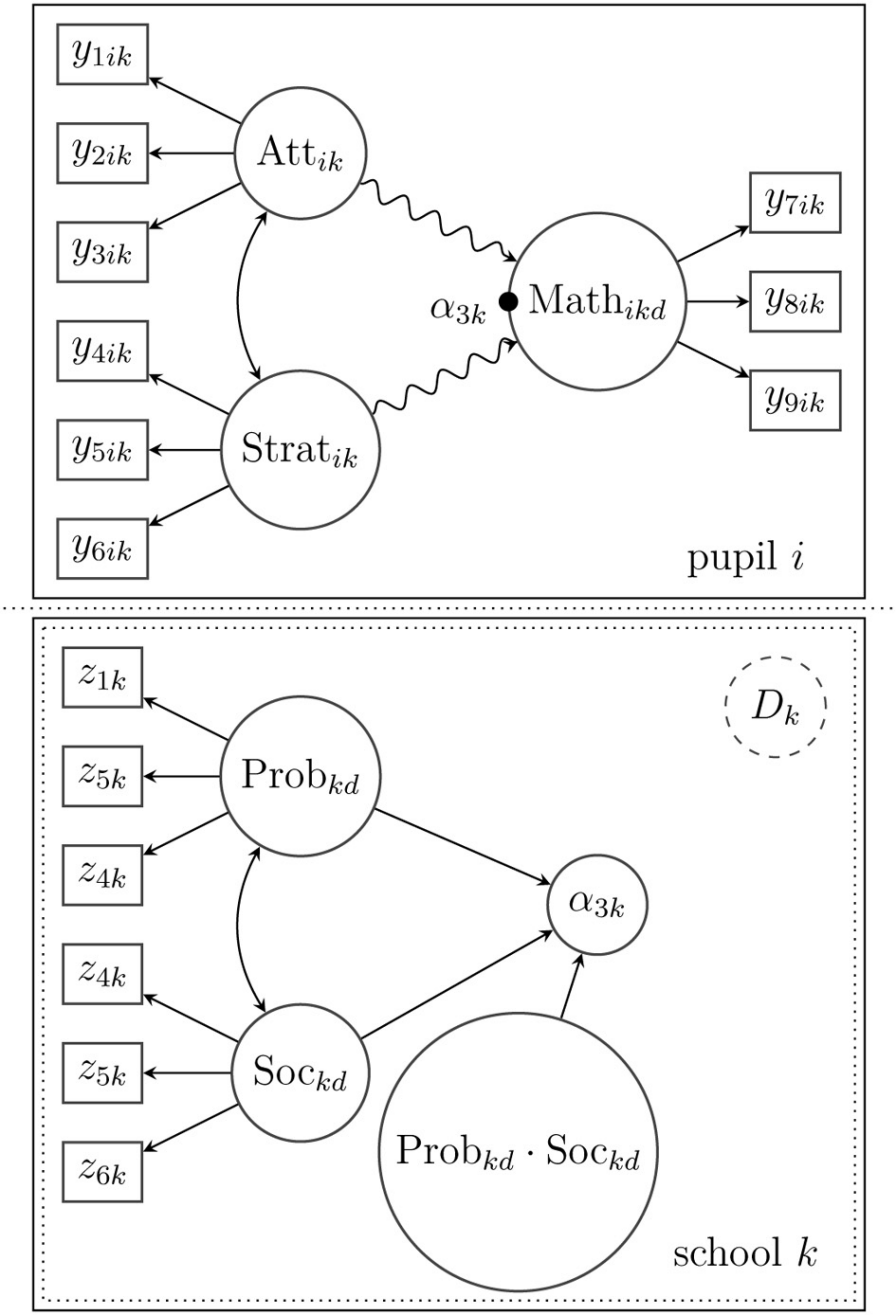
**Structural models and measurement models on the within-level (Level 1) and between-level (Level 2)**. On Level 1, the math skill (Math) of a pupil *i* is predicted by his/her general attitude toward reading (Att) and his/her experienced teaching strategies (Strat). The snake-type arrows indicate a flexible spline approximation of the latent variable relationship. On Level 2, the average math skills of pupils (latent intercept α_3*k*_) in school *k* are explained by a nonlinear interaction between structural problems in the school (Prob) and the school's social environment (Soc). The non-normality of the latent predictors is approximated by a mixture distribution.

In the following, we will provide the specified measurement and structural equations for the model. For reasons of clarity, we restricted the subscripts (*k*, *c* or *d*) in the model formulation to those model parameters that actually depended on the latent classes or the Level-2 model. Figure 6 presents a diagram of the model and its parameters.

#### 6.1.1. Structural models

The Level-1 structural model [cf. Equation (4)] for the *i*-th pupil in school *k* was given by

(21) $$\begin{array}{l} \eta_{1ik}=\alpha_{k}+B_{1}F_{1}(\eta_{1ik})+\zeta_{1ik}\\ \left(\begin{array}{c} {\text{Att}}_{ik}\\ {\text{Strat}}_{ik}\\ {\text{Math}}_{ik} \end{array}\right)=\left(\begin{array}{c} \alpha_{1}\\ \alpha_{2}\\ \alpha_{3k} \end{array}\right)+\left(\begin{array}{cc} 0 & 0\\ 0 & 0\\ \beta_{1} & \beta_{2} \end{array}\right)\;\cdot \;\left(\begin{array}{c} F_{11}({\text{Att}}_{ik})\\ F_{12}({\text{Strat}}_{ik}) \end{array}\right)+\left(\begin{array}{c} \zeta_{11ik}\\ \zeta_{12ik}\\ \zeta_{13ik} \end{array}\right) \end{array}$$

where *F*_11_ and *F*_22_ both defined a latent cubic spline model with two knots at ξ_1_ = 2, ξ_2_ = 3 that approximated the (curvilinear) relationships between the variables (e.g., Hastie et al., 2009):

(22) $$\begin{array}{l} \beta_{1}F_{11}({\text{Att}}_{ik})=\;\beta_{11}{\text{Att}}_{ik}+\beta_{12}{\text{Att}}_{ik}^{2}+\beta_{13}{\text{Att}}_{ik}^{3}\\ \;+\beta_{14}{\left({\text{Att}}_{ik}-\xi_{1}\right)}_{+}^{3}+\beta_{15}{\left({\text{Att}}_{ik}-\xi_{2}\right)}_{+}^{3}\\ \beta_{2}F_{12}({\text{Strat}}_{ik})=\;\beta_{21}{\text{Strat}}_{ik}+\beta_{22}{\text{Strat}}_{ik}^{2}+\beta_{23}{\text{Strat}}_{ik}^{3}\\ \;+\beta_{24}{\left({\text{Strat}}_{ik}-\xi_{1}\right)}_{+}^{3}+\beta_{25}{\left({\text{Strat}}_{ik}-\xi_{2}\right)}_{+}^{3}. \end{array}$$

Only the latent intercept α_3*k*_ was assumed to vary across schools. The Level-2 structural model [cf. Equation (6)] for school *k* was given by

(23) $$\begin{array}{l} \eta_{2k}{|}_{D_{k}\;=\;d}=\;\mu_{d}+B_{2}F_{2}(\eta_{2kd})+\zeta_{2k}\\ \left(\begin{array}{c} {\text{Prob}}_{k}{|}_{D_{k}\;=\;d}\\ {\text{Soc}}_{k}{|}_{D_{k}\;=\;d}\\ \alpha_{3k} \end{array}\right)=\left(\begin{array}{c} \mu_{1d}\\ \mu_{2d}\\ \mu_{3} \end{array}\right)+\left(\begin{array}{ccc} 0 & 0 & 0\\ 0 & 0 & 0\\ \beta_{3} & \beta_{4} & \beta_{5} \end{array}\right)\\ \;\cdot \;\left(\begin{array}{c} {\text{Prob}}_{kd}\\ {\text{Soc}}_{kd}\\ {\text{Prob}}_{kd}\cdot {\text{Soc}}_{kd} \end{array}\right)+\left(\begin{array}{c} \zeta_{21k}\\ \zeta_{22k}\\ \zeta_{23k} \end{array}\right) \end{array}$$

with **η**_2*kd*_ = (Prob_*kd*_, Soc_*kd*_, α_3*k*_)′ and *F*_2_(**η**_2*kd*_) = (Prob_*kd*_, Soc_*kd*_, Prob_*kd*_ · Soc_*kd*_)′. The product term Prob_*kd*_ · Soc_*kd*_ implemented the interaction effect of the structural problems in school and the social environment. Because the non-normal distributions of the latent predictors were approximated by a mixture distribution, their expectations μ_1*d*_ and μ_2*d*_ were assumed to vary across the unobserved mixtures (Kelava and Nagengast, 2012).

#### 6.1.2. Measurement models

For each of the latent variables between nine and 13 items were available; they were aggregated to three indicator variables for each latent variable (item parcels) for didactic purposes. It was assumed that the indicator variables were continuously distributed, resulting in an identity link function in the measurement model (y^*^_ik_ = **y**_*ik*_ and z^*^_k_ = **z**_*k*_, respectively).

On Level 1, the measurement model for pupil *i* in the *k*-th school [cf. Equation (3)] was given by

(24) $$y_{ik}=\nu_{1}+\Lambda_{1}f_{1}(\eta_{1ik})+\epsilon_{1ik}$$

where *f*_1_(**η**_1*ik*_) = (Att_*ik*_, Strat_*ik*_, Math_*ik*_)′.

On Level 2, the measurement model [cf. Equation (7)] was given by

(25) $$z_{k}{|}_{\;D_{k}\;=\;d}\;=\nu_{2}+\Lambda_{2}f_{2}(\eta_{2kd})+\epsilon_{2k}$$

where *f*_2_(**η**_2*kd*_) = (Prob_*kd*_, Soc_*kd*_)′. The factor loading matrices **Λ**_1_ and **Λ**_2_ were formulated with a simple structure (i.e., each item loaded on only one latent variable). The residual variables **ϵ**_1*ik*_ and **ϵ**_2*ik*_ were assumed to be mutually uncorrelated and normally distributed with zero mean vectors and (diagonal) covariance matrices **Θ**_1_ and **Θ**_2_, respectively.

#### 6.1.3. Parameter constraints and identification

Besides employing the standard identification constraints for structural equation models, we restricted the measurement model parameters and the structural model parameters to be the same across schools except for the latent intercept α_3*k*_. Due to the invariance of the measurement models for the latent predictors on Levels 1 and 2, in Equations (24) and (25) the non-linear effects in the polynomial spline model and the interaction effect in Equations (22) and (23) were identified. For the mixture model, we fit two latent classes (*D*_*k*_ = 1, 2).

### 6.2. Model estimation

To keep this example as simple as possible, missing data were assumed to be missing at random, and this was accounted for directly in the analysis by applying the Gibbs sampler (Gelman et al., 2004). The analysis of the latent multilevel model was implemented by using the R-project software (R Core Team, 2013) and the OpenBugs package (Lunn et al., 2009). Syntax for the empirical example can be obtained upon request from the authors.

#### 6.2.1. Starting values and prior selection

Starting values for the measurement model parameters were based on the prior analyses conducted in Mplus Muthén and Muthén (1998–2010) for separate parts of the model. Informative priors were then selected in accordance with recommendations by Gelman et al. (2004) as well as Kelava and Nagengast (2012).

#### 6.2.2. Bayesian analysis

For the analysis, three chains with 100,000 iterations each were generated. The first 75,000 iterations (burn in) were then discarded. As proposed by Gelman (1996), convergence of the estimation procedure was achieved when all (EPSR Estimated Potential Scale Reduction; Gelman, 1996) values were below 1.2, which occurred after about 60,000 iterations (see the Supplementary Material, Figure S1). Trace plots also indicated good convergence (see the Supplementary Material, Figure S2). Means, standard errors, *t*-values, and percentiles of the posterior distributions of the parameter estimates based on the last 25,000 iterations are reported in the next subsection.

### 6.3. Results

We will summarize the main results in this subsection. Detailed results for the estimated multilevel model are presented in Table 1. In the measurement models, the factor loadings were all significant and positive, thus indicating that the latent constructs were measured reliably.

**Table 1:** **Mean parameter estimates, standard errors, *t*-values, and 2.5, 50.0, and 97.5% percentiles**.

	$\bar{\hat{\theta }}$	***SE***	***t*-value**	**Percentiles**		
				**2.5%**	**50.0%**	**97.5%**
**LEVEL-1 PARAMETERS**						
**Intercepts**						
ν_121_	−1.078	0.076	−14.190	−1.229	−1.076	−0.933
ν_131_	−0.409	0.072	−5.709	−0.557	−0.408	−0.275
ν_152_	0.411	0.118	3.484	0.173	0.414	0.633
ν_162_	−0.419	0.175	−2.399	−0.769	−0.411	−0.089
ν_183_	0.058	0.018	3.317	0.023	0.058	0.092
ν_193_	0.340	0.016	21.069	0.308	0.340	0.372
**Factor loadings**						
λ_121_	1.141	0.026	43.986	1.091	1.141	1.192
λ_131_	0.997	0.024	40.814	0.951	0.996	1.047
λ_152_	0.687	0.043	15.864	0.605	0.686	0.774
λ_162_	1.213	0.064	18.849	1.091	1.210	1.343
λ_183_	0.754	0.030	25.279	0.696	0.754	0.814
λ_193_	0.553	0.027	20.437	0.501	0.553	0.607
**Path coefficients**						
β_11_	0.005	0.161	0.031	−0.312	0.004	0.320
β_12_	0.009	0.117	0.079	−0.221	0.009	0.237
β_13_	−0.005	0.029	−0.174	−0.061	−0.005	0.052
β_14_	0.046	0.068	0.678	−0.088	0.046	0.180
β_15_	−0.164	0.104	−1.580	−0.367	−0.165	0.037
β_21_	−0.070	0.192	−0.366	−0.453	−0.060	0.287
β_22_	0.079	0.080	0.992	−0.073	0.077	0.239
β_23_	−0.017	0.012	−1.449	−0.040	−0.017	0.006
β_24_	0.007	0.016	0.443	−0.025	0.007	0.039
β_25_	0.018	0.029	0.620	−0.040	0.018	0.073
**Means/intercepts of latent variables**						
α_1_	2.856	0.022	129.972	2.813	2.856	2.899
α_2_	2.700	0.019	143.505	2.663	2.700	2.737
**(Co)variances of latent variables**						
ψ_111_	0.506	0.025	20.357	0.459	0.506	0.556
ψ_121_	0.072	0.012	5.866	0.048	0.072	0.097
ψ_122_	0.250	0.019	13.076	0.215	0.250	0.291
ψ_133_	0.041	0.003	15.847	0.036	0.041	0.046
**Residual variances**						
θ_111_	0.147	0.009	16.124	0.129	0.147	0.165
θ_122_	0.198	0.012	16.607	0.176	0.198	0.222
θ_133_	0.212	0.011	19.288	0.191	0.212	0.234
θ_144_	0.212	0.014	14.708	0.184	0.213	0.241
θ_155_	0.323	0.014	22.999	0.297	0.323	0.352
θ_166_	0.219	0.019	11.251	0.181	0.219	0.257
θ_177_	0.066	0.003	19.760	0.059	0.066	0.072
θ_188_	0.047	0.002	20.197	0.042	0.047	0.052
θ_199_	0.049	0.002	23.364	0.045	0.049	0.053
**LEVEL-2 PARAMETERS**						
**Latent class probabilities**						
*P* (*D* = 1)	0.532	0.255	2.082	0.069	0.537	0.955
*P* (*D* = 2)	0.468	0.255	1.835	0.045	0.463	0.931
**Intercepts**						
ν_221_	0.759	0.415	1.829	−0.063	0.767	1.550
ν_231_	0.603	0.277	2.180	0.048	0.603	1.143
ν_252_	−0.024	0.184	−0.129	−0.391	−0.020	0.331
ν_262_	0.279	0.179	1.556	−0.077	0.281	0.614
**Factor loadings**						
λ_221_	1.029	0.206	4.999	0.635	1.027	1.439
λ_231_	0.700	0.137	5.113	0.434	0.699	0.970
λ_252_	1.002	0.091	11.071	0.828	1.001	1.180
λ_262_	0.794	0.088	8.992	0.629	0.793	0.969
**Path coefficients**						
β_3_	0.558	0.101	5.512	0.381	0.550	0.776
β_4_	0.442	0.108	4.072	0.261	0.432	0.680
β_5_	−0.289	0.053	−5.483	−0.405	−0.285	−0.199
**Means/intercepts of latent variables**						
μ_11_	1.921	0.099	19.408	1.685	1.935	2.076
μ_21_	1.938	0.081	24.063	1.739	1.950	2.062
μ_12_	2.107	0.123	17.088	1.931	2.084	2.424
μ_22_	2.091	0.104	20.076	1.955	2.071	2.367
μ_3_	−0.365	0.162	−2.248	−0.686	−0.361	−0.059
**(Co)variances of latent variables**						
ψ_211_	0.291	0.049	5.959	0.207	0.287	0.396
ψ_221_	0.007	0.024	0.304	−0.038	0.007	0.055
ψ_222_	0.239	0.030	7.986	0.186	0.237	0.305
ψ_233_	0.051	0.005	11.169	0.042	0.050	0.060
**Residual variances**						
θ_211_	0.415	0.059	6.970	0.305	0.413	0.539
θ_222_	0.723	0.098	7.412	0.543	0.718	0.927
θ_233_	0.366	0.046	7.898	0.280	0.364	0.461
θ_244_	0.183	0.022	8.204	0.143	0.182	0.230
θ_255_	0.130	0.017	7.763	0.100	0.129	0.165
θ_266_	0.176	0.020	8.940	0.141	0.175	0.217

**Figure 7:**
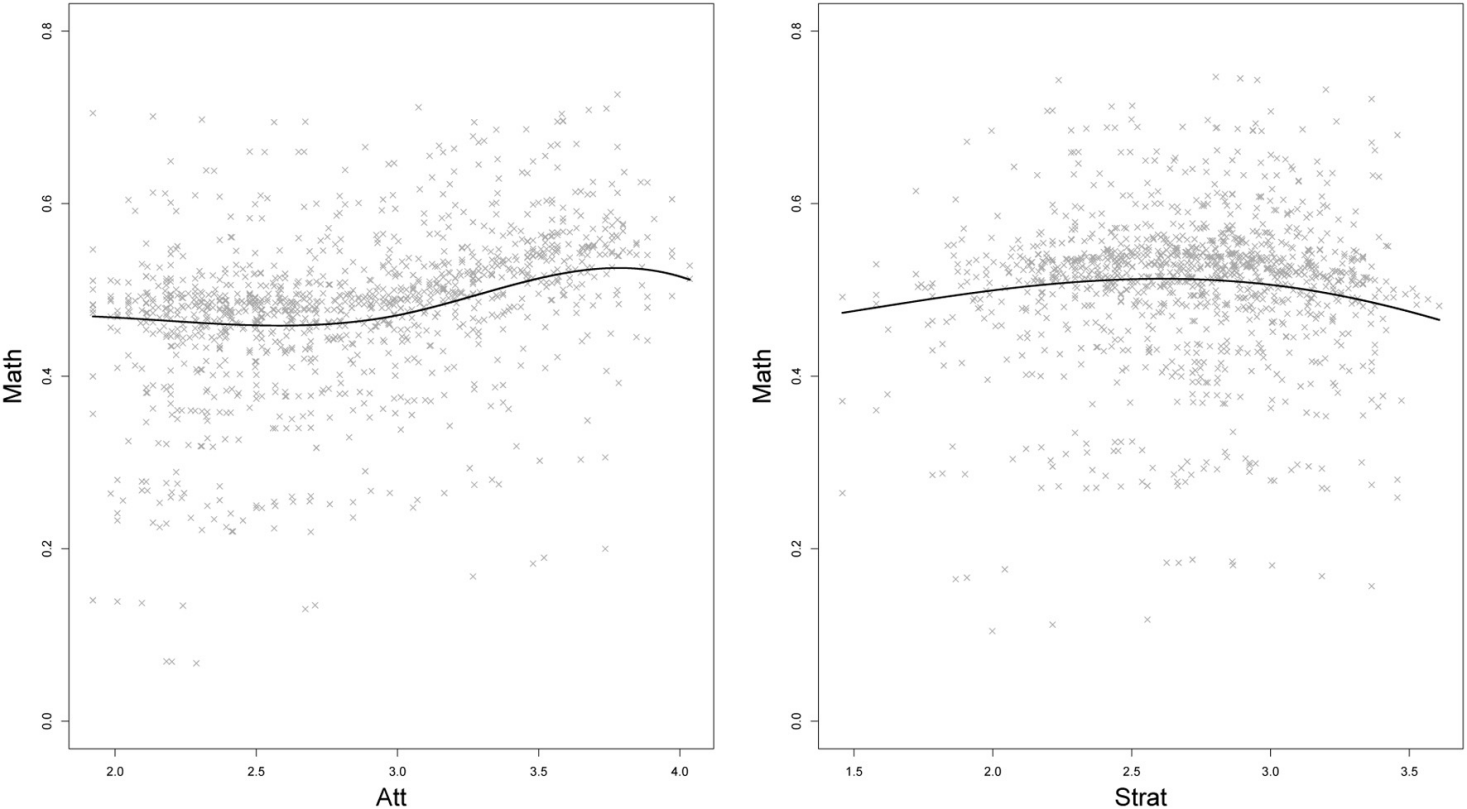
**Semiparametric Level-1 relationships between *pupils' math skills* (Math) and their *general attitude toward reading* (Att; left), and Math and *experienced teaching strategies* (Strat; right)**. The gray crosses indicate the predicted slope with a predicted school-specific random intercept; the black line indicates the predicted Math score for the mean random intercept.

The results for the semiparametric approximation of the true relationships between the Level-1 latent variables Att, Strat, and Math are illustrated in Figure 7. The relationship between Math and Att resembled a cubic relationship; the subjects' Math scores slowly increased with increasing Att scores, whereby a stronger increase was found for Att scores greater than 3 and a slight decrease for Att scores greater than 4. The relationship between Strat and Math seemed to be slightly quadratic with the highest Math scores for medium Strat scores.

In order to test the hypotheses on the cubic relationship for Att and the quadratic relationship for Strat^4^, we estimated a model that changed Equation (22) to **β**_1_*F*(Att_*ik*_) = β_11_Att_*ik*_ + β_12_Att^2^_*ik*_ + β_13_Att^3^_*ik*_ and **β**_2_*F*_12_(Strat_*ik*_) = β_21_ Strat_*ik*_ + β_22_Strat^2^_*ik*_. Results for the structural parameters on the within-level can be found in Table 2. The parametric cubic relationship for Att was not significant ($\hat{\beta }$_13_ = 0.003, *p* = 0.745 for the cubic effect and $\hat{\beta }$_11_ = − 0.045, *p* = 0.723 for the linear effect). The attitude toward reading did not significantly predict the math ability. The parametric model for Strat indicated a significant negative quadratic relationship ($\hat{\beta }$_22_ = −0.034, *p* = 0.037). This indicated that pupils' math skills were highest for those subjects who rated the experienced teaching strategies as average.

**Table 2:** **Mean parameter estimates, standard errors, *t*-values, and 2.5, 50.0, and 97.5% percentiles for the parametric model (cubic relationship for Att and quadratic relationship for Strat) on Level 1**.

	$\bar{\hat{\theta }}$	***SE***	***t*-value**	**Percentiles**		
				**2.5%**	**50.0%**	**97.5%**
**PATH COEFFICIENTS**						
β_11_	−0.045	0.139	−0.324	−0.307	−0.045	0.232
β_12_	0.005	0.055	0.097	−0.105	0.006	0.112
β_13_	0.003	0.007	0.354	−0.012	0.003	0.017
β_21_	0.154	0.082	1.877	0.000	0.157	0.329
β_22_	−0.034	0.016	−2.086	−0.067	−0.034	−0.003

**Figure 8:**
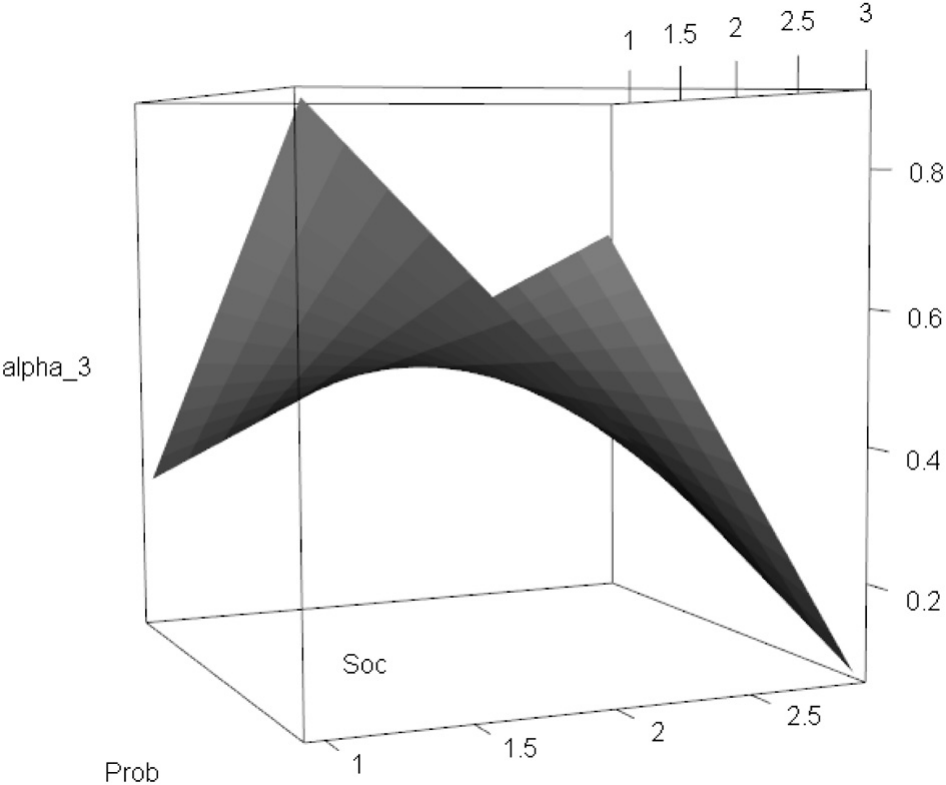
**Between-level: Three-dimensional illustration of the relationship between school problems (Prob), social problems (Soc), and the random intercept α_3*k*_ of Math**.

On Level 2, the random intercept factor α_3*k*_ had a significant negative intercept ($\hat{\mu }$_3_ = −0.365, *p* = 0.024) and an unexplained variance across schools of $\hat{\psi }$_233_ = 0.051. The linear effects of the predictors were significant with $\hat{\beta }$_3_ = 0.558 (*p* < 0.001) for school problems (Prob) and $\hat{\beta }$_4_ = 0.442 (*p* < 0.001) for social problems (Soc). The interaction effect was significant and negative with $\hat{\beta }$_5_ = −0.289 (*p* < 0.001). Figure 8 illustrates the complex non-linear association between Prob, Soc, and the random intercept α_3*k*_. The expected math level of a school with an average score on school and social problems was about 0.5 (*E*[α_3_|*Prob* = *Prob*, *Soc* = *Soc*] = 0.461); the expected math level was higher in schools for which one of the problems was above average and the other was below average; and the math level decreased rapidly when both problems increased together.

**Figure 9:**
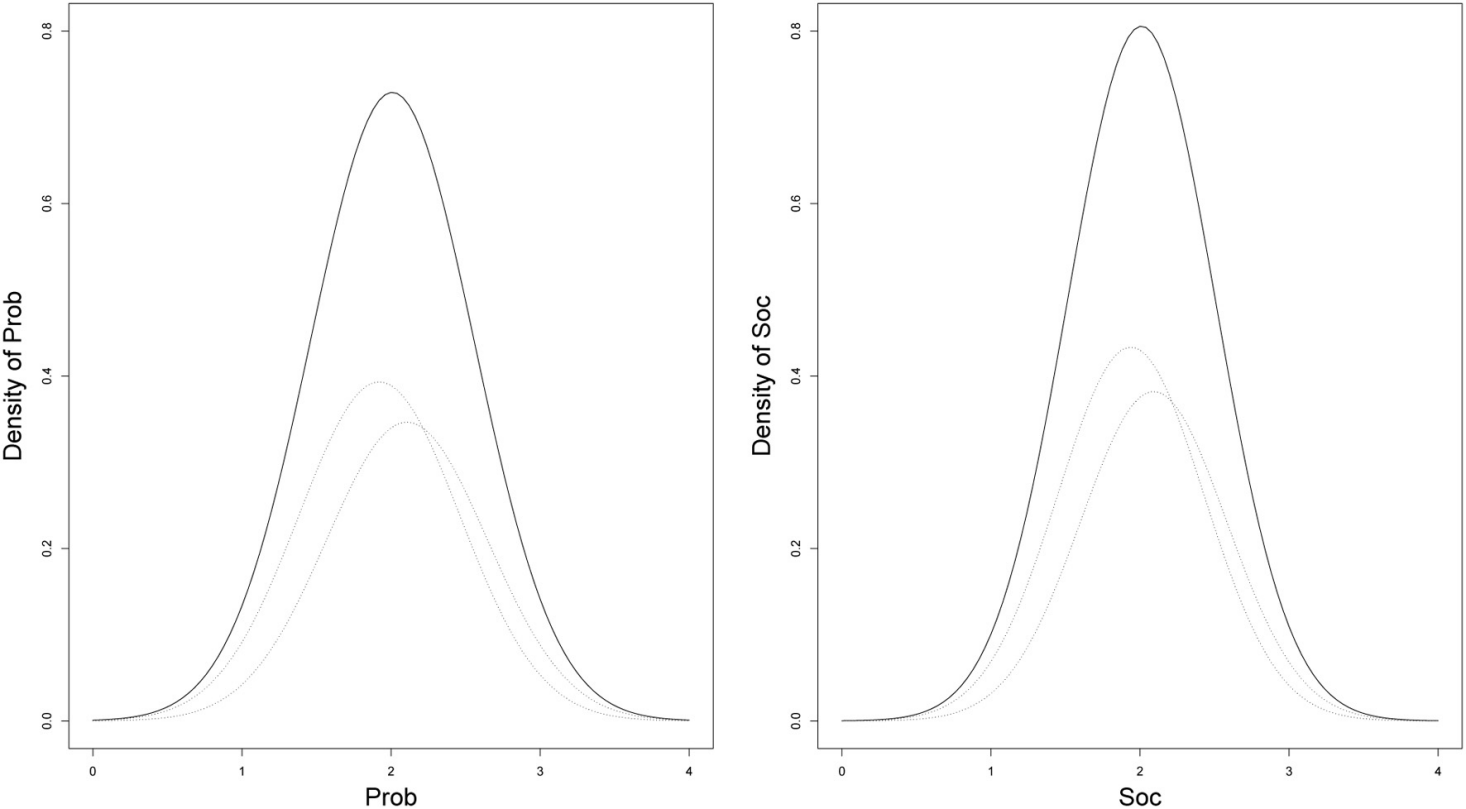
**Predicted slightly non-normal densities of the Level-2 predictors Prob and Soc obtained from a two-class solution**.

Finally, the results of the mixture model for the Level-2 predictors are illustrated in Figure 9. As can be inferred from Figure 9, the distribution of the latent variables was slightly non-normal. In this empirical example, the means of the latent variables in the two classes were marginally different (with means of about $\hat{\mu }$_11_ ≈ $\hat{\mu }$_21_ ≈ 1.9 in Class 1 and $\hat{\mu }$_12_ ≈ $\hat{\mu }$_22_ ≈ 2.1 in Class 2). Additional analyses may reveal the necessity to increase or decrease the number of latent classes (e.g., using the DIC). Here, the DIC was 14,780 for a model including the mixtures and 14,770 for a model without the mixture distribution. This indicates that a mixture may not have been necessary in this case.

## 7. Discussion

In this article, we presented a generalized non-linear multilevel structural equation mixture model (GNM-SEMM) framework. A key characteristic its ability to specify non-linear functional relationships between outcome variables on one side and latent predictors or manifest covariates on the other side by using semiparametric regression functions (e.g., splines; Freund and Hoppe, 2007; Hastie et al., 2009). This feature is given for both levels, the within and between (cluster) levels of nested data structures. Given that (multilevel) latent variable modeling frameworks are typically linear (Bollen, 1989; van der Linden and Hambleton, 1997; Rabe-Hesketh et al., 2004; Muthén and Asparouhov, 2011), the relaxation of the linearity assumption is a step forward toward a more realistic approximation of a non-linear world. It thus extends the hitherto available multilevel modeling frameworks.

A second key characteristic is the ability to specify latent mixture distributions on both levels. As in recent semiparametric latent variables approaches (e.g., Bauer and Curran, 2004; Bauer, 2005; Kelava et al., 2014), this allows for an approximation of non-normally distributed latent predictor variables for a thorough introduction with regard to manifest variables, see McLachlan and Peel (2000). Again, the relaxation of a typical assumption that can be found in most applications of latent variable modeling allows for a more precise modeling of relationships for heterogeneous populations or distributions.

A third key characteristic of the proposed approach is that it is flexible enough to specify a large number of special cases. For example, it offers the ability to approximate a non-normal distribution using mixture modeling and provides an easy way to interpret the parametric functional form of the latent variable relationship. As another example, it is possible to specify a non-linear latent variable relationship in one subpopulation but not in the other. The same is true for different levels. If functional forms of the relationships are unknown, semiparametric approximations of these relationships are also possible using mixtures.

Taken together, these properties are desirable. Nevertheless, the identification and estimation of the models is a general issue. Additional assumptions have to be introduced as was exemplified in the sections before (see Level-1 section on the measurement model). Fortunately, these assumptions are standard identification assumptions in latent mixture, latent (non)linear, and (semi)parametric modeling, but researchers should be careful when specifying models. For example, multiple intercepts in spline models might lead to identification issues. However, the wide range of specifiable models offers a variety of adaptable estimators that could be applied from a theoretical standpoint. Bayesian MCMC, Newton-type algorithms, and adapted EM-Algorithms are just a few examples.

In this paper, we also used a substantive example from educational science. A complex model was applied to data from the large-scale PISA study (Organisation for Economic Co-Operation and Development, 2010) illustrating several conditions that may occur in empirical data. First, an a priori unknown curvilinear relationship between the latent variables was identified on Level 1 using a semiparametric latent spline model. Second, the proposed mixture part on Level 2 could be used to control for the potential non-normality of the latent Level-2 predictors. In this example, only a slight indication of non-normality was visible. The model may have also been extended to include a mixture model on Level 1. Third, on Level 2 a latent random intercept modeled a school-dependent math skill, which allowed us to account for the clustering of the data. The random intercept was predicted by a latent non-linear interaction model. The model may be extended further, for example, to test the linearity assumption on Level 2 of the relationship between the latent variables apart from the interaction effect. Other random effects could also be included. In any case, the specification of these effects should be theory-driven.

Finally, we want to mention two important considerations. The proposed model should be viewed as a general framework that includes a variety of different possible models. A model that includes all aspects as presented in the model section would be highly parameterized and may overfit the data. In each empirical situation, we recommend that the actual applied model be restricted to a simpler model that allows for an adequate but parsimonious representation of the data. A decision concerning the necessity to include different parts of the model depends on the hypothesized model (e.g., random factor loadings in a confirmatory factor model or a latent spline to predict a latent slope in the structural model) and on model comparisons. In the Bayesian framework, Bayesian indices/information criteria for model selection (e.g., the deviance information criterion, DIC: Spiegelhalter et al., 2002; Celeux et al., 2006; or the Bayes factor, Bernardo and Smith, 1994) are the primary model fit indices, although they only allow only for a model comparison to be made, and they are not absolute fit indices. In general, for (both frequentist and Bayesian) non-linear models there are no absolute fit indices (Klein and Schermelleh-Engel, 2010). Hence, a top-down (or bottom-up) strategy using information criteria may be a viable way to improve the model (i.e., to restrict the model to its necessary parts). An illustration of such a strategy for multilevel models in general can be found, for example, in West et al. (2007).

Furthermore, we did not show how to implement the presented framework with statistical software. In this article, a Bayesian estimator was applied and implemented in OpenBugs, thus allowing us to analyze a complete but specific semiparametric non-linear multilevel model. Future research should improve this implementation so that it will be feasibly available within standard statistical latent variable software (e.g., Mplus) that can be directly applied to different models by the substantive researcher.

### Conflict of interest statement

The authors declare that the research was conducted in the absence of any commercial or financial relationships that could be construed as a potential conflict of interest.

## Footnotes

^1^In SEMM linear models are estimated within several latent classes. Non-linear relationships between two variables are modeled by the parameter estimates for the linear effects that change in size across the (finite number of) latent classes.

^2^An exception is the special case in which the coefficient matrix **B** = **0**: that is, for confirmatory factor models.

^3^The ICC was 0.407 for the manifest variable, which was the sum of all Math items.

^4^A direct inference with regard to a parametric relationships, including a linear relationship, based on the parameter estimates for the spline model (e.g., β_11_) is not straightforward (Cox et al., 1988; Cox and Koh, 1989; Zhang and Lin, 2003; Liu and Wang, 2004). In general, an additional model that can actually test a parametric hypothesis seems to be a viable procedure (Azzalini and Bowman, 1993).
