# Supplementary material for: A general non-linear multilevel structural equation mixture model
Source: Front Psychol. 2014 Jul 18;5:748. doi: 10.3389/fpsyg.2014.00748 (PMC4102910; doi:10.3389/fpsyg.2014.00748)
Supplement: Supplementary file 1 [file Presentation1.PDF]

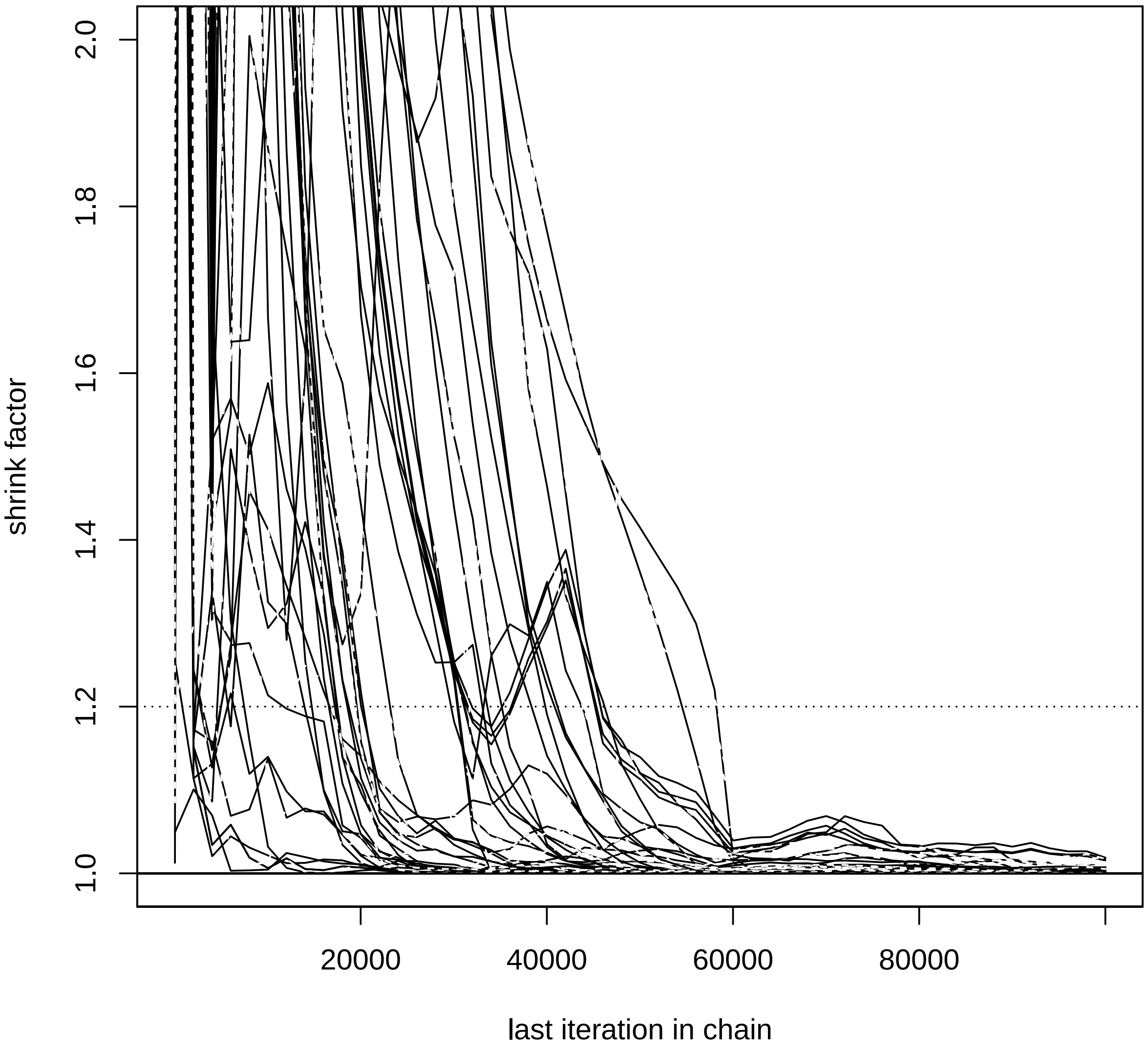

Figure 1. EPSR values for the parameters. The horizontal dotted line indicates the cutoff of EPSR = 1.2; the solid lines indicate the median estimates. Convergence was achieved after about 60,000 iterations.

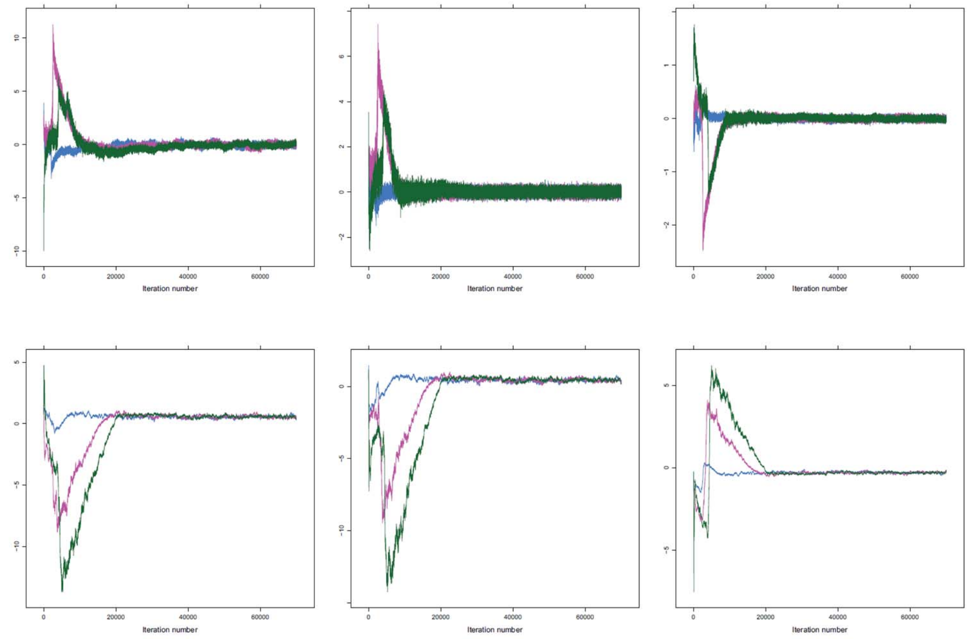

Figure 2. Trace plots for three selected parameters on Level 1 ($\beta_{11} - \beta_{13}$, first row) and on Level 2 ($\beta_3 - \beta_5$, second row).
